# Focal nodular hyperplasia of the liver: an unusual association with diabetes mellitus in a child and review of literature

**DOI:** 10.1186/1824-7288-36-41

**Published:** 2010-05-26

**Authors:** Piero Farruggia, Rita Alaggio, Francesca Cardella, Serena Tropia, Antonino Trizzino, Francesca Ferrara, Paolo D'Angelo

**Affiliations:** 1 Unit of Pediatric Hematology and Oncology, "G. Di Cristina" Children's Hospital, Palermo, Italy; 2 Section of Pathology, Department of Oncologic Sciences, University of Padua, Italy; 3 Department of Pediatrics, University of Palermo, Italy

## Abstract

Hepatic hemangioma, adenoma and focal nodular hyperplasia are the most frequent benign lesions of the liver, but they are all infrequent among pediatric population. The reports of focal nodular hyperplasia in children have recently increased in number, with many cases associated to drug intake, particularly to chemotherapy. We here describe, to our knowledge, the first case of focal nodular hyperplasia in association with diabetes mellitus in childhood.

## Background

The focal nodular hyperplasia (FNH) first described by Edmondson in 1956, accounts for 8% of primary hepatic tumors in adults and less than 2% in children. Nonetheless, its evidence in pediatric population is increasing with more than eighty cases described in the last 5 years. The pathogenesis of FNH is largely unknown. Evidences of polyclonality in different DNA studies exclude a neoplastic nature of the lesion and further support the hypothesis of a possible reactive hyperplastic response of liver cells to local vessel abnormality. Chemotherapy and/or radiotherapy may be associated with development of FNH and, as clarified by a case-control study, also smoke may play a pathogenetic role [1]. The association with estroprogestinic therapies is still debated [2].

## Case presentation

**Figure 1:**
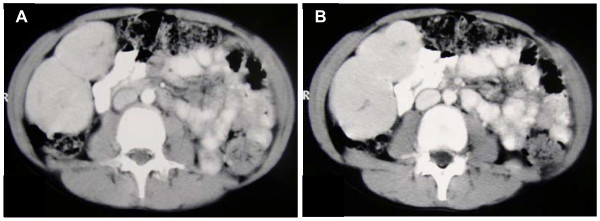
**Abdomen CT scan showed two masses, with an evidence of tumor capsule, that, after contrast injection, were hyperdense in early (arterial) phase (A) and isodense, compared to the remaining liver parenchyma, in venous phase (B); it also highlighted a central hypodense "scar"**.

A.L., male, suffering from type 1 diabetes mellitus since he was 7 years old; the disease was metabolically compensated with stable insulin doses. At the age of 12 he was admitted for abdominal pain and a voluminous mass in right hypochondria was found; he was in good general conditions (32 kg of weight) and remaining physical examination was normal. His insulin requests were: regular insulin 5 IU before breakfast, 10 IU before lunch and 6 IU before dinner; NPH insulin 16 IU at bedtime. The liver ultrasound scan showed, at the VI liver segment, two hypo-isoechogenous oval lesions whose diameters were, respectively, 83 × 43 mm and 77 × 38 mm. CT scan (Fig. 1A, B) confirmed the presence of two masses, with an evidence of tumor capsule, that, after contrast injection, were hyperdense in early (arterial) phase and isodense, compared to the remaining liver parenchyma, in venous phase; it also highlighted a central hypodense "scar".

**Figure 2:**
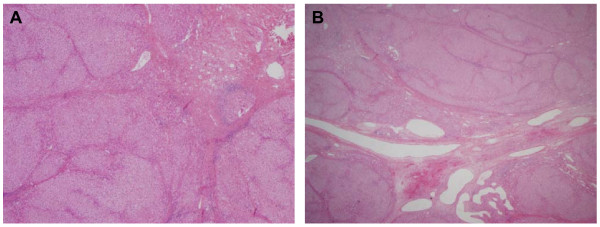
**Both lesions (A, B) were classic FNH showing a nodular hyperplastic parenchyma with a typical central fibrous scar, containing a proliferation of small bile ducts, irregular tortuous arteries with thickened walls, veins and capillaries**. A discrete inflammatory infiltrate filled the fibrous septa surrounding the hepatocytic nodules.

Routine blood tests were all normal and the detection of serological markers of HAV, HBV, HCV, HIV and CMV was negative: CEA and αFP were also negative. On the basis of the mild abdominal pain, and also for a formal request of parents, the child underwent surgical intervention: the two masses were completely removed. The gross appearance was that of lobulated, well circumscribed masses, lacking a fibrous pseudocapsule and showing a central scar with fibrous septa running to the periphery and partially demarcating nodular structures. At histologic analysis, both lesions were classic FNH showing a nodular hyperplastic parenchyma with a typical central fibrous scar, containing a proliferation of small bile ducts, irregular tortuous arteries with thickened walls, veins and capillaries. A discrete inflammatory infiltrate filled the fibrous septa surrounding the hepatocytic nodules (Fig. 2A, B). The child had a fully satisfactory post-surgery course in terms of metabolic compensation. The outbreak of fever justified a treatment with broad-spectrum antibiotic. After 7 years the child is in good health and no recurrence has been noted.

## Discussion

FNH is very rare in pediatric population with an age prevalence in children 7-8 years old, although some cases are diagnosed in early childhood or even in the prenatal period [3]. The female sex is predominant with a M/F ratio of less than 1/10 in one of the largest series [4]. The cause is unknown although the hypothesis that obstruction of hepatic vessels or abnormal vascularization could account for FNH is suggested by the reported association with clinical and anatomic findings like hypoplasia or agenesis of the portal vein, vascular malformations, hemangioma and vascular dysplasia, Budd-Chiari syndrome and hereditary hemorrhagic telangiectasia [5,6]. Moreover, recent studies reported angiopoietin-1 (Ang-1) gene and angiopoietin-2 (Ang-2) involvement in the regulation of vasculogenesis in FNH, with Ang-1/Ang-2 ratio increase in FNH compared with normal liver and benign or malignant hepatocellular neoplasms [7]. Lastly, not only it is proved that FNH is more frequent in females and after oral contraceptive but also there are evidences that estrogens may induce hepatic angiogenesis in the rat [8]. All in all, it is possible to assume that pathogenetic mechanisms may be related to an hyperplastic response to hemodynamic disturbances due to local factors (eg, vascular abnormalities or local venous thrombosis with subsequent arteriovenous shunts) or systemic factors (eg, oral contraceptives and angiogenic molecules) [5]. As it is known that diabetes typically is a risk factor for arterial stiffening and vein thrombosis it is possible to speculate that the association of diabetes and FNH could be not completely incidental [9]. It is a bit intriguing to note that HNF1α, a tumour suppressor gene, whose abnormal expression could enhance tumorigenesis, is inactivated in 35-50% of all liver adenomas, which are, like FNH, much more frequent in women taking oral contraceptives: moreover germline mutation of HNF1α can be associated with maturity onset diabetes of the young 3 (MODY3). Taking together this data can resume a final model where anomalies in gluconeogenesis, glycolysis and lipogenesis (all typical of diabetes), female sex and oral contraceptives may contribute to hemodynamic anomalies and to the subsequent development of benign neoplasms like FNH and adenoma.

The majority (70-90%) of FNH at presentation is asymptomatic and the most common way that the disease is discovered is when, during an occasional physical examination, hepatomegaly or a palpatory abdominal mass are detected. More rarely the disease is diagnosed after the occurrence of abdominal pain, even though this event appears not exceptional in pediatric age. Almost ever the liver function tests are normal. The lesion is more often unique, but about 8% of cases may show multiple nodules, up to 30. The diameter of lesions is extremely variable, from less than 1 cm to more than 15 cm but usually is less than 5 cm.

Some cases of associations with specific disorders or therapies, have been published:

1. Glycogen storage disease type I: described up to now about 10 cases.

2. Infrequent cases reported in association to adrenal cancer, adrenal pseudocyst or Cushing's syndrome.

3. Rare and doubtful cases in association with drepanocytosis.

4. Three cases reported after portoenterostomy for biliary atresia.

5. Some cases reported after intake of drugs such as clomifene, busulfan and especially androgens.

6. A plenty of pediatric cases detected after chemotherapy as well as after Hematopoietic Stem Cell Transplantation (HSCT) [10].

7. Only five cases of association with diabetes mellitus have been reported until now: three cases were mentioned into a series of 28 Swedish patients, another one was a German 62-year old woman with long-term diabetes and the fifth was an Australian patient [11-13]. They were all adult with typical type 2 diabetes.

Different histological variants of FNH are recognized: the classic, the most frequent and corresponding to the present case, the not classic, including FNH with cytologic atypia, and mixed hyperplastic and adenomatous FNH. The so-called telangiectatic FNH is now considered a variant of adenoma [14].

Classic FNH is characterized by hepatocellular trabeculae forming nodules separated by fibrous septae radiating from a central fibrous scar. The lesion is generally well circumscribed, without a capsule but surrounded by a rim of compressed parenchyma. The central scar may be absent or inconspicuous in many cases. The evidence of a typical vascular component is an important feature, probably related to the vascular malformative pathogenesis of the lesion.

With modern equipment, the diagnosis is easy in the majority of cases:

1. Ultrasound. Typical FNH is a mass with variable echogenicity and the typical hyperechogenic central scar. Moreover, with the doppler, the presence of areas of hypervascularization is appreciated in about 50% of the cases. The flow is predominantly arteriosus type, with high-speed and low resistance and irradiation from the center to the periphery. With the contrast enhanced ultrasound the typical pattern is characterized by an high arterial and portal venous enhancement.

2. CT scan. Hyperintense lesion after administration of contrast; there is often an immediate contrast enhancement, mostly isodense, with high initial attenuation. There is also the ability to detect, when it is present, the hypodense area of the central scar. So it is possible to say that typical FNH lesions have homogeneous enhancement in hepatic arterial phase, presence of central scar, and delayed enhancement, while the atypical FNH, more frequent in male sex, may show heterogeneous immediate enhancement, absence of central scar, or presence of pseudocapsule [4].

3. MRI. The enhancement after injection of contrast tends to be early, intense and homogeneous and the lesion appears more frequently as isointense or hypointense in T1 and isointense or slightly hyperintense in T2, even if atypical presentations are possible.

Needle-biopsy or open air biopsy are necessary when the radiological investigations are doubtful, above all in case of absence of the central scar, and not rarely the differential diagnosis from other nodular lesions of liver may be difficult. The differential diagnosis includes different nodular lesions of the liver.

Nodular regenerative hyperplasia (NRH) is a disease characterized by multiple nodules composed by hepatocytes, without a fibrous tissue or central scar [15]. It is often associated to patients suffering of inflammatory bowel disease treated with thioguanine or azathioprine or systemic diseases, above all connective tissue diseases, such as LES, Polyarteritis nodosa, Antiphospholipid Syndrome, Rheumatoid Arthritis or Felty's Syndrome. The rare pediatric cases are mostly in association with the congenital absence of portal vein (sometimes complicated by heart disease or multicystic kidney dysplasia). CT presentation is really different from FNH, as there are multiple hypodense lesions with poor or absent enhancement after contrast administration [15].

The typical imaging showing anechogenic and regular profile of the mass at ultrasound, easily recognize cystic lesions: however CT and MRI may be necessary in selected cases.

In haemangiomas a doppler ultrasound test is diagnostic almost ever. At CT scan the contrast enhancement proceeds typically from center to periphery. Nonetheless in adults and, exceptionally, in children, it is possible to have a concomitance of FNH and haemangioma.

Radiological findings play a key role also in the diagnosis of Adenoma (HCA). At ultrasound there is not the central scar and the doppler effect is predominantly venous type with either continuous or pulsatile peripheral flow: typically absent is the portal venous enhancement at the contrast enhanced ultrasound. MRI and CT may also highlight spots of hemorrhage and necrosis. The differential diagnosis is crucial, because the adenoma is at risk of breaking, haemorrhage, and malignant transformation. Surprisingly there are uncommon cases of simultaneous detection of adenoma and FNH. Moreover, the ductular proliferation may be minimal in some FNH at histological examination, making the differential diagnosis difficult [14].

Hepatoblastoma, the most frequent liver tumor in childhood, is almost always associated to α-fetoprotein (α-FP) increase and there is not the central scar at CT scan. Similarly to what happen in adenoma, there are exceptional cases of concomitant presence of hepatoblastoma and FNH. The hepatocellular carcinoma, exceptional in pediatric population and often associated with α-FP increase, at the CT scan shows an hypodense lesion with a less prompt contrast enhancement than FNH and, at MRI, an heterogeneous initial enhancement after contrast injection.

The natural evolution of FNH is unpredictable even if the mass, in about 2/3 of cases, remains stable and in about 1/3-1/4 of cases shows a gradual spontaneous improvement as far as a complete remission; it is rare an increase in number as well as in size [9].

The recent studies in molecular biology have confirmed that FNH is not a pre-neoplastic lesion: the tissue parenchimal organization is pretty the same of usual liver tissue and, moreover, even though in some cases a clonal origin of FNH nodules have been demonstrated, until now no somatic mutation in the β-catenin gene or in the other genes implicated in the hepatocellular adenoma (where a malignant transformation is possible) have been discovered [5,16]. Moreover the gene expression profile in FNH shows an activation in β-catenin pathway limited to the enlarged perivenous areas and completely different with respect to the other hepatocellular benign lesions [17].

About the management the first step is, of course, the stop of oral contraceptive. Considering the body of evidence that FNH doesn't undergo malignant transformation and that there are only sporadic cases followed by spontaneous rupture and consequent abdominal bleeding, we agree with the opinion that in asymptomatic cases it is opportune a careful follow-up with an ultrasound scan every 6-12 months, and that elective surgery has probably to be limited to the patients suffering of abdominal pain or with a voluminous or growing mass [5]. It is worth to note that recurrences are very rare after surgical removal. When surgical resection is impossible due to patient factors or location and size of the tumor, selective arterial embolization should be considered. Finally it is possible that novel drugs or other radiological therapies like radio frequency ablation will be available in the future.

We think that reporting children with FNH may be helpful to provide to clinicians data useful for the differential diagnosis process and for the therapeutic decisions: remarkably this is the sixth case of FNH associated to diabetes, the first, at our knowledge, in a child.

## Consent

Written informed consent was obtained from the parents of the patient for publication of this case report and accompanying images. A copy of the written consent is available for review by the Editor-in-Chief of this journal.

## Competing interests

The authors declare that they have no competing interests.

## Authors' contributions

PF, FC, FF and PD revised the clinical history and drafted the manuscript. RA carried out histological examination, provided the pictures of pathologic findings and contributed in preparing the manuscript. ST and AT provided useful contribution in the revision of literature and participated in writing discussion. All authors read and approved the final manuscript.
